# Malaria prevalence and patients’ knowledge, attitude, and preventive practices toward the disease in the Jawi District, Awi Zone, Northwest Ethiopia

**DOI:** 10.3389/fpara.2025.1535306

**Published:** 2025-02-04

**Authors:** Mekete Damen, Damtew Bekele, Fikru Gashaw

**Affiliations:** ^1^ Department of Biology, College of Natural and Computational Sciences, Kotebe University of Education, Addis Ababa, Ethiopia; ^2^ Department of Biology, College of Natural and Computational Sciences, Ambo University, Ambo, Ethiopia

**Keywords:** awareness, Jawi, malaria, *Plasmodium falciparum*, prevalence

## Abstract

**Background:**

Malaria is the most important parasitic illness causing morbidity and mortality with high prevalence in tropical regions.

**Objective:**

This study was aimed at evaluating the 7-year malaria trend and community awareness at Jawi Health Center and primary Hospital in Northwest Ethiopia.

**Methods:**

A retrospective and cross-sectional or prospective design were used for the study. The data was analyzed using SPSS version 22 software. The findings were considered significant at P < 0.05.

**Results:**

Among 62,624 blood films between 2015 and 2021 at Jawi Health Center, 40.9% were positive. *Plasmodium falciparum* accounted for 85.8%. Women had more mixed infections (*P. falciparum* and *P. vivax*) (X^2^ = 8.9, df = 2, P = 0.011) than men. A greater proportion (20.6%) of malaria cases was observed within the under 5 years age group and the number of malaria cases was higher in September, October, and June. The overall prevalence of malaria was found to be 25.2% and June had the highest proportion (75.6%). In total, 335 (80.9%) respondents recognized mosquito bites as the cause and fever (50%) as a clinical symptom of malaria. More than half of the respondents (60.1%) never sleep under mosquito nets.

**Conclusion:**

Thus, these findings have substantial implications for the trend of malaria prevalence and patient awareness of the disease which support the existing malaria control efforts.

## Introduction

Malaria is a protozoan infection of red blood cells transmitted by the bite of a female anopheles mosquito and causes massive morbidity and mortality. More than 90% of the malaria burden occurs in sub-Saharan Africa (SSA), mainly affecting young children in rural areas with limited access to health services. It also affects the poorest and most vulnerable communities due to their lack of access to effective services for prevention, diagnosis, and treatment. Of the five most common species of *Plasmodium* that infect humans, the vast majority of deaths in SSA are caused by *Plasmodium falciparum*, while *Plasmodium vivax*, *Plasmodium malaria, Plasmodium ovale*, and *Plasmodium knowlesi* generally cause milder forms of the disease (World malaria report, 2018; Herrador et al., 2019).

In Ethiopia, malaria is a major challenge for both public health and socio-economic development. Three-quarters of its landmass is considered endemic for malaria, putting 68% of the total population more at risk of the infection (EPHI (Ethiopian Public Health Institute), 2016). This protozoan infection (by *Plasmodium* species) was responsible for 14% of outpatient visits and 9% of admissions in the country in 2009/2010 (FMOH, 2011). In 2016, there were an estimated 2,927,266 new malaria cases and 4,782 deaths (Girum et al., 2019). Varying topographical and climatic features of the country contribute to the seasonal and unstable malaria transmission pattern which is usually characterized by frequent focal and cyclical widespread epidemics.

The transmission of malaria peaks biannually from September to December and April to May, coinciding with the major harvesting seasons (FMOH, 2010). *P. falciparum* and *P. vivax* are the two dominant species in Ethiopia, which account for 60% and 40% of malaria cases, respectively. However, the proportion varies among geographical settings and seasons. In the lowlands and during the major malaria transmission season, *P. falciparum* is much more common while *P. vivax* is more common at higher altitudes and during the dry season. The disease primarily occurs at altitudes below 2,000 meters above sea level in the country (Hailu et al., 2017).

Different studies have tried to assess knowledge, perceptions, and behaviors toward malaria among communities in different parts of the country. These studies have demonstrated the communities’ level of understanding and revealed some misconceptions related to signs and symptoms, mode of transmission, treatment, and prevention control of malaria. In a study from the Tigray region, Ethiopia, nearly half of the respondents (48.8%) recognized mosquitoes as a cause of malaria (Paulander et al., 2009). It was also observed that misconceptions about the causes and transmissions of malaria were very common, where exposure to cold weather, hunger, and drinking dirty water were mentioned as causes of malaria (Aderaw and Gedefaw, 2013). In contrast, findings of a study conducted on two indigenous populations of Bangladesh revealed superficial knowledge of malaria transmission, prevention, and treatment by the respondents (Ahmed et al., 2009).

Studies showed that each year, more than 300 million malaria cases and up to 3 million deaths occur throughout the world. Of these, over 80% of malaria deaths occur in Africa, with Asia and Eastern Europe accounting for less than 15% (World Health Organization, 2015). Although the climatic conditions of Jawi and its surroundings are suitable for mosquito breeding, *Plasmodium* infection around the town is not known. Thus, this study was conducted to assess the trend of malaria and patients’ awareness of the disease in selected health centers in the Jawi District, Northwest Ethiopia.

## Materials and methods

### Description of the study area

**Figure 1:**
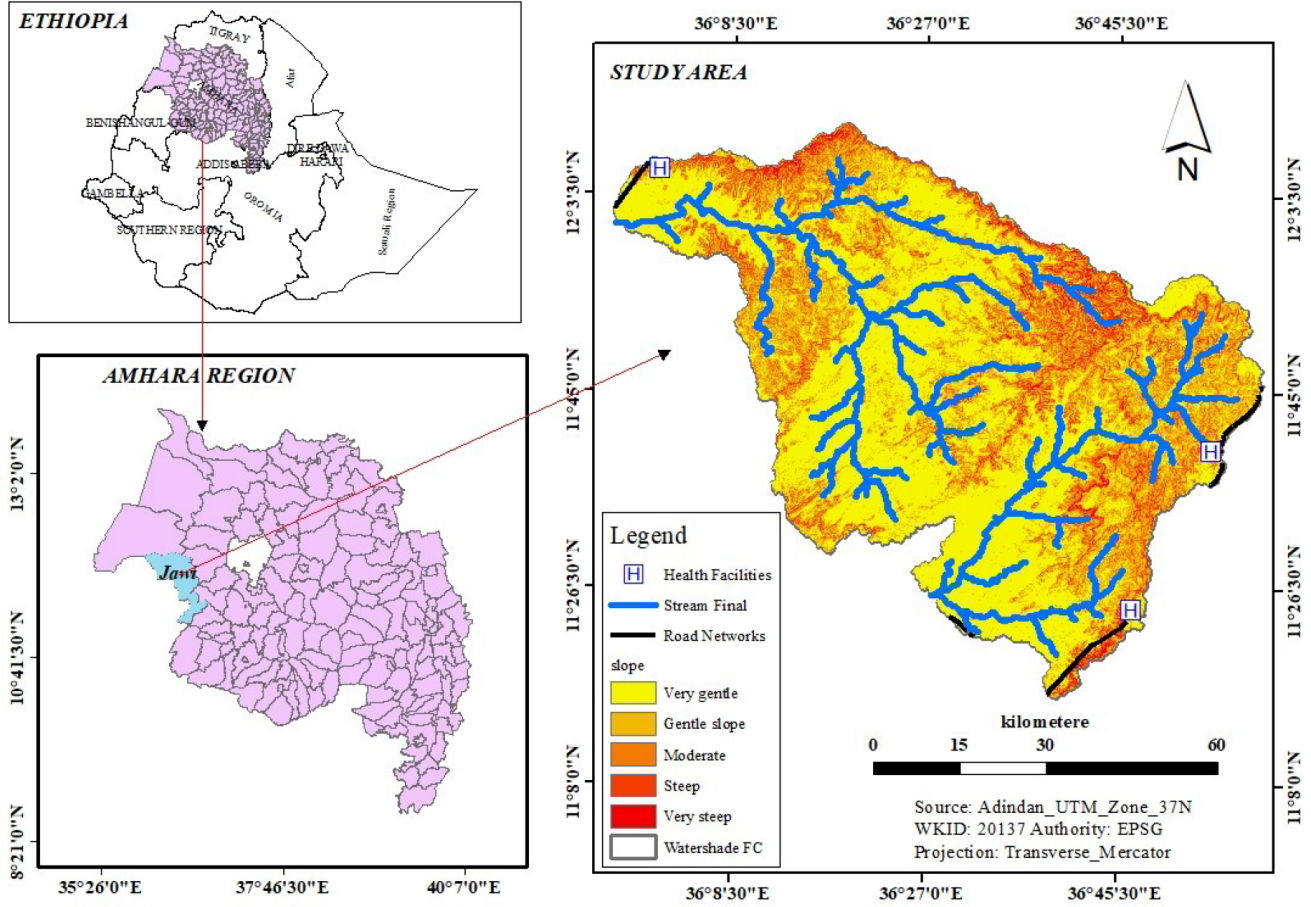
Map of the study area.

The study was conducted in the Jawi District, Awi Zone, Amhara National Regional State of Ethiopia. The district is located 714 km northwest of Addis Ababa and 280 km from the capital city (Bahir Dar) of the Amhara region. The district has a total area of 515,400 km^2^ and it shares geographical borders with the Alefa and Quara Districts in the north, the Achefer and Dangilla Districts in the east, and the Benishangul-Gumuz region in the south and west. The district is located within the geographical locations of 10°38’ to 11°30’ N latitude and 36° to 37° E longitude (**Figure 1**). There are two major ethnic groups in the district (Amhara and Agew), with an additional small number from Oromia. It has an elevation ranging from 653 to 2,245 meters above sea level with an average temperature of approximately 38.5°C and an average annual rainfall of approximately 1,200–1,225mm (Sitotaw et al., 2019).

### Study design and period

A health facility-based retrospective study design was employed over a seven-year period (2015 to 2021) and a cross-sectional study over 4 months (March to June in 2022) to determine malaria prevalence in the Jawi District. The awareness of the patients of the disease was further assessed using a structured questionnaire.

### Study population, data collection, and analysis

All clinically suspected malaria patients at the Jawi Primary Hospital and Jawi Health Center were incorporated into the study. Based on the data availability in the health institutes, the patient’s registration book was used to determine the trend of malaria for the last 7 years (2015 to 2021) at the Jawi Health Center and infections due to the *Plasmodium* parasite for 4 months (March to June 2022) during the study period in Jawi Primary Hospital. A data extraction sheet was prepared to collect the patients’ socio-demographic characteristics. A structured questionnaire with open and closed-ended questions was designed and completed by all malaria-positive patients (414) to assess their knowledge, attitude, and practice (KAP) concerning the cause, mode of transmission, and preventive methods. The questionnaire was prepared in English and then translated into the local language (Amharic) by an expert who is fluent in both languages to maintain its consistency. It was administered by health professionals if the respondents were not able to read and write.

Data analysis was made using Statistical Package for the Social Sciences (SPSS) software version 22. Descriptive statistics were run to measure the frequencies and percentages of the variables. The prevalence of malaria based on age, sex, month, year, and species of the malaria parasite was analyzed. To compare the trend of malaria prevalence among sex, age groups, and months, the chi-square test was used. A statistically significant rate was considered at p < 0.05.

### Ethical clearance

The study proposal was reviewed at the Department of Biology, Kotebe Metropolitan University and a permission letter was obtained for the study (Ref.No KUE/Biol/078/14). Based on this, an additional permission letter was also obtained from the Jawi administrative health office to carry out the study. Discussions were held with the health institute’s administrative body and laboratory workers about the objective of this study, and a permission letter was obtained to utilize blood for the stated purpose. Moreover, the objectives of the study were described to patients suspected of having malaria and informed consent was obtained.

## Results

### Malaria prevalence by year, month, sex, and age

**Figure 2:**
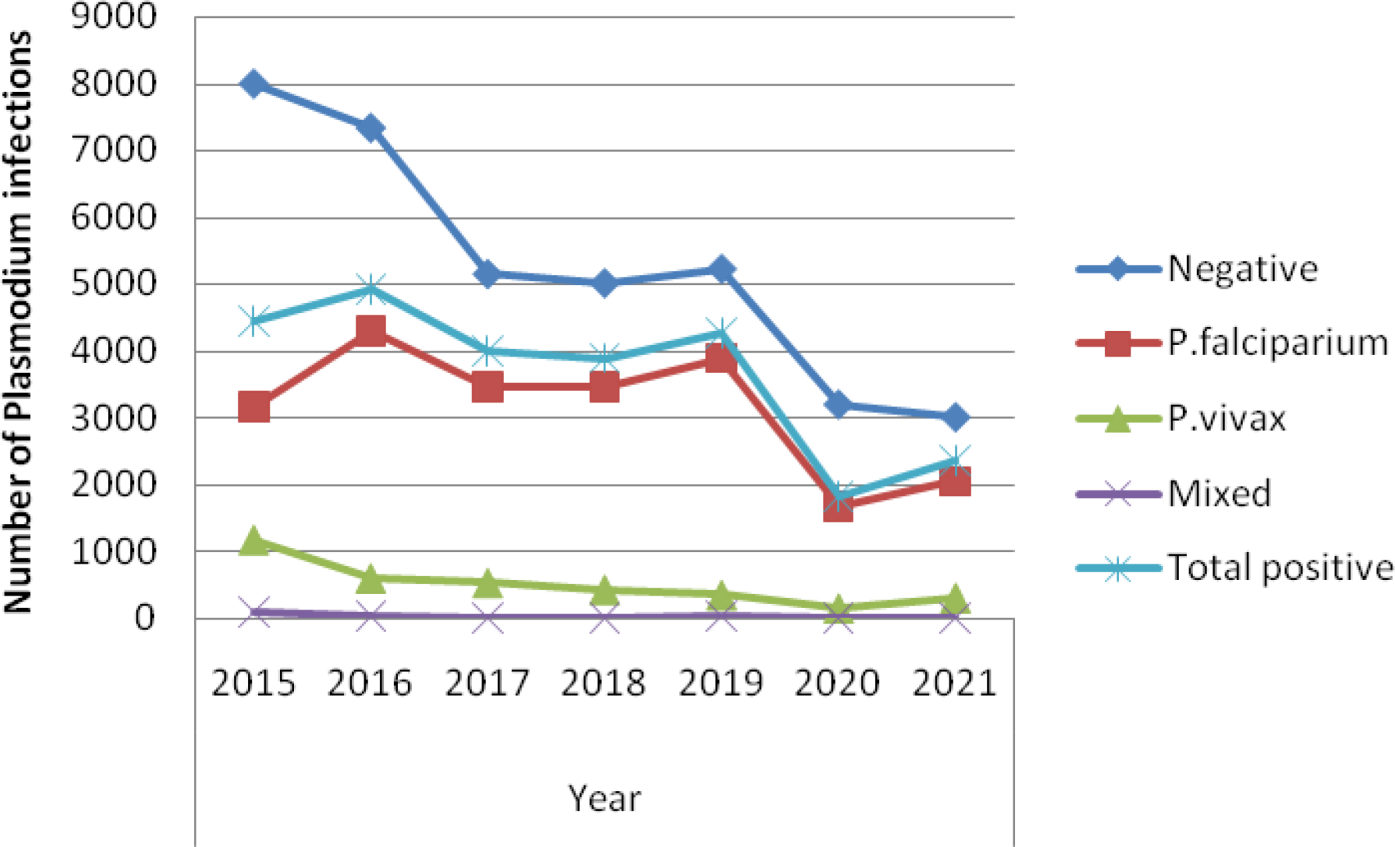
Trend of *Plasmodium* infections by year (2015 to 2021) among patients tested in the Jawi Health Center, Awi Zone, Northwest Ethiopia.

A total of 62,624 blood films were diagnosed based on a health institute’s smear test for malaria within the 7 years (2015 to 2021), of which 25,638 (40.9%) were positive for the disease. Among the infected individuals, *P. falciparum* infection accounted for 22,008 (85.8%), *P. vivax* accounted for 3,448(13.4%), and mixed infection accounted for 182 (0.7%) cases. The most malaria infections, 4,912 (19.2%), were recorded in 2016, and the least, 1,822 (7.1%), in 2020 (**Figure 2**) in which the association of malaria patients with the 7 consecutive years of the study period had a statistically significant difference (X^2^ = 1041.5, df, = 12, p = 0.000).

### Monthly distribution of malaria in Jawi Health Centre

Malaria cases peaked in September (2,574, 10.0%), October (3,360, 13.1%), and June (3,028, 11.8%), while April (1,411, 5.5%) recorded the lowest number of cases (N=25,740). The association between malaria and monthly infections had a statistically significant difference (X^2^ = 138.6, df, = 22, p = 0.000). At the species level, *P. falciparum* infections peaked in October at 3008 (13.7%) and June at 2665 (12.1%), whereas *P. vivax* infections peaked in September at 400(11.6%) and mixed infections in May at 30 (16.5%). In contrast, the lowest incidence of *P. falciparum*, *P. vivax*, and mixed infections was recorded in April with 1,194 (5.4%), 210 (6.1%), and 7 (3.8%), respectively.

### Plasmodium species infections with s*ex g*roups

Male patients were more affected (13,883, 54.2%) by malaria than female patients within the study period and the difference was statistically significant (X^2^ = 8.9, df = 2, p = 0.011). Infections with *P. falciparum* were also the most dominant ones throughout the 7-year study period.

### Plasmodium species infections with age groups

Malaria infection was recorded among all age groups with a statistically significant association between its burden and age groups (X^2^ = 433.2, df, 15, p= 0.000). Those under 5 and those between 18 and 23 were the most affected age groups with 5,286 (20.6%) and 5,246 (20.5%), respectively. The mean and standard deviation for the infected age group were 17.23 and 13.08 years, respectively. *P. falciparum* was the predominant parasite in all age groups with a prevalence rate of 85.8%, followed by *P. vivax* with a prevalence rate of 13.4%, but mixed infection had the lowest prevalence rate in all age groups at 0.7%. *P. falciparum* had the most cases within the age group of 18–23 years, followed by the under-5 age group.

### Overview of the prospective study on malaria infection

A total of 1,644 suspected patients were examined between March and June 2022 of which 1,230 were negative cases and 414 (25.2%) were positive. There were 186 (44.9%) male and 228 (55.1%) female infected cases. The majority of positive malaria cases were female each month, with *P. falciparum, P. vivax*, and mixed infections accounting for 282 (68.1%), 130 (31.4%), and 2 (0.5%), respectively. The degree of infection was greater for *P. falciparum* than for *P. vivax* or mixed infections (X^2^ = 9.385, df = 6, P = 0.153). More malaria cases were also identified in the month of June than in the rest (**Table 1**).

**Table 1:** Number and proportion by sex of *Plasmodium* infection prevalence among patients who visited Jawi Primary Hospital from March to June 2022.

Month	Sex	Negative	*P. falciparium* positive (%)	*P. vivax* positive (%)	Mixed infection positive (%)	Total no. (%)
March	Male	96	25 (54.3)	10 (34.5)	0 (0)	35 (46.7)
	Female	95	21(45.6)	19 (65.5)	0 (0)	40 (53.3)
	Total	191	46 (100)	29 (100)	0 (0)	75 (1000)
April	Male	88	23 (45.1)	7 (46.7)	1(100)	31(46.2)
	Female	135	28 (54.9)	8 (53.3)	0 (0)	36 (53.7)
	Total	223	51(100)	15 (100)	1(100)	67 (100)
May	Male	174	26 (39.4)	20 (50)	0 (0)	46 (43.4)
	Female	203	40 (60.6)	20 (50)	0 (0)	60 (56.6)
	Total	377	66 (100)	40 (100)	0 (0)	106 (100)
June	Male	211	55 (46.2)	19 (41.3)	0 (0)	64 (38.6
	Female	227	64 (53.8)	27 (58,7)	1(100)	92 (55.4
	Total	438	119 (100)	46 (100)	1(100)	166 (100
Total	Male	569	128 (45.4)	56 (43.1)	1(50)	186 (44.9)
	Female	661	154 (54.6)	74 (56.9)	1 (50)	228 (55.1)

### Assessment of knowledge, attitude, and preventive practice toward malaria

A total of 414 questionnaires were completed with a response rate of 100% (**Table 2**). A majority of the respondents were under the age of 7 years (107, 25.8%) and more participants were female (55.1%) than male (44.9%). In addition, 290 (17.7%) were from rural areas and 124 (7.5%) were from urban areas.

**Table 2:** Socio-demographic characteristics of the patients (n=414).

Variables	Category	Frequency (%)
Age	Under 7	107 (25.80)
	8 – 15	53 (12.80)
	16 – 23	98 (23.70)
	24 – 31	82 (19.80)
	32 – 39	28 (6.80)
	40 – 47	22 (5.30)
	48 – 55	9 (2.20)
	56 – 63	5 (1.20)
	64 – 71	5 (1.2)
	72 – 79	4 (1.00)
	80 and above	1 (.20)
Sex	Male	186 (44.90)
	Female	228 (55.10)
Marital status	Single	229 (55.30)
	Married	165 (39.90)
	Divorced	11 (2.70)
	Widowed	9 (2.20)
Family size	< 5	294 (71.00)
	≥ 5	120 (29.00)
Educational status	Illiterate	245 (59.20)
	Only read and write	68 (16.4)
	Grade 1-8	48 (11.60)
	Grade 9-12	22 (5.30)
	More than 12	31 (7.50)
Occupational status	Government employment	43 (10.40)
	Farmer	118 (28.50)
	Merchant	39 (9.40)
	Daily laborer	60 (14.50)
	Housewife	81(19.60)
Living Place	Urban	124 (30.0)
	Rural	290 (70.00)

A higher *P. falciparum* malaria prevalence was observed in the 16–23 years age group. In the case of *P. vivax*, a high infection rate was recorded among the less than 7 years old age group.

### Knowledge of malaria prevention methods

Patients were asked if they had ever heard of malaria and all of them responded in the positive. All the study participants also agreed that malaria is one of the major health problems in the study area. The major symptoms of malaria were reported as fever (205, 49.5%) and chills and shivering (123, 29.7%). Most study participants (335, 80.9%) were aware that malaria is transmitted via a bite of a mosquito infected with *Plasmodium*, mainly at night (373,90.1%) (**Table 3**).

**Table 3:** Knowledge of patients on malaria prevention methods.

Items	Category	Frequency (%)
What are the signs and symptoms of malaria?	Fever	205 (49.5)
	Chills and shivering	123 (29.7)
	Headache	45 (10.9)
	Loss of appetite	21 (5.1)
	Back or joint pain	5 (1.2)
	Vomiting	5 (1.2)
	Weakness	10 (2.4)
Can malaria be transmitted from person to person?	Yes	368 (88.9)
	No	17 (4.1)
	Do not know	29 (7.0)
How malaria is transmitted?	Mosquito bite	335 (80.9)
	Patient contact	14 (3.4)
	Hot environment	24 (5.8)
	Poor environmental hygiene	41 (9.9)
Where do mosquitoes breed?	Marshy area	110 (26.6)
	Stagnant water	266 (64.3)
	Discarded materials	23 (5.6)
When do mosquitoes mostly bite?	Days	11(2.7)
	Night	373 (90.1)
	I do not know	25 (6.0)
	Equally day and night	5 (1.2)
What preventive method do you know?	Anti-mosquito spray in the house	99 (23.9)
	Sleep in a mosquito net	177 (42.8)
	Drainage of stagnant water	88 (21.3)
	Clean bushes	20 (4.8)
	Visit the health center	27 (6.5)
What might be the reasons for not using any protective methods?	Not affordable	43 (10.4)
	Not available	189 (45.7)
	Not aware of its use	20 (4.8)
	Economic problem	162 (39.1)

Regarding knowledge of interventions for indoor and outdoor prevention and vector control, 99 (23.9%) used anti-mosquito spray inside the home, 20 (4.8%) cleaned the bushes around the house, 88 (21.3%) drained stagnant water, and 27(6.5%) visited the health center when they became ill to prevent malaria. Furthermore, 42.8% of respondents cited using a bed net while sleeping as a malaria prevention measure.

### Attitude of patients towards malaria prevention methods

The majority of patients (52.9%) agreed and strongly agreed (44.0%) that malaria is a serious and life-threatening disease. More than half of the respondents also agreed (60.6%) and strongly agreed (22.5%) that malaria can be transmitted from one person to another as with other communicable diseases. A large proportion of the participants responded that avoiding mosquito bites plays a key role in the prevention of malaria. More than 400 (96.6%) of the respondents agreed and strongly agreed that malaria is a greater risk for children and pregnant women. The vast majority of participants, 260 (62.8%), agreed that working and sleeping overnight in the garden or forest might lead to a higher risk of getting malaria (**Table 4**).

**Table 4:** Patients’ attitude towards malaria prevention methods.

Questions	Category	Frequency	Percentage (%)
Malaria is a serious and life-threatening disease.	Agree	219	52.9
	Strongly agree	182	44.0
	Disagree	13	3.1
	Strongly disagree	0	0.0
Malaria can transmit from person to person as with other communicable diseases.	Agree	251	60.6
	Strongly agree	93	2.2
	Disagree	66	15.9
	Strongly disagree	4	1.0
The best way to prevent getting malaria is to avoid getting mosquito bites.	Agree	239	57.7
	Strongly agree	168	40.6
	Disagree	7	1.7
	Strongly disagree	0	0.0
Sleeping under a mosquito net at night is one way to prevent getting malaria.	Agree	242	58.5
	Strongly agree	162	39.1
	Disagree	10	2.4
	Strongly disagree	0	0.0
Malaria is a greater risk for children, pregnant women, and persons with malnutrition.	Agree	107	25.8
	Strongly agree	293	70.8
	Disagree	14	3.4
	Strongly disagree	0	0.0
Working and sleeping overnight in the garden or forest might put one at greater risk of getting malaria.	Agree	185	44.7
	Strongly agree	75	18.1
	Disagree	152	36.7
	Strongly disagree	2	0.4
I might be at a greater risk of getting malaria if I do not fully cover all of my body during the night.	Agree	235	56.8
	Strongly agree	42	10.1
	Disagree	131	31.6
	Strongly disagree	6	1.4

### Practices of respondents’ malaria prevention methods

A majority of the study participants (249, 60.1%) responded that they did not sleep in mosquito nets whilst 153 (37.0%) and 12 (2.9%) participants reported sleeping under mosquito nets sometimes and every time, respectively. More than half (257, 62.1%) of the study participants reported that their family members never slept under insecticide-treated nets, but 155 (37.4%) and 2 (0.4%) respondents reported sleeping under treated mosquito nets sometimes and every time, respectively, and 339 (81.9%) did not have a habit of checking the presence of holes/repair in the mosquito nets to avoid mosquito bites. Most of the participants never used mosquito-repellent coils in their houses. Nobody had any practice of constantly draining standing water where *Anopheles* mosquitoes may breed. Of the total respondents, 220 (53.1%) reported that they sometimes drain stagnant water or moist areas around their residences. Among the total number of participants, 341 (82.4%) wore clothes that covered all of their body during nighttime (**Table 5**).

**Table 5:** Practices of patients regarding malaria prevention methods.

Items	Category	Frequency	Percentage (%)
How often do you sleep in a mosquito net?	Every time	12	2.9
	Sometimes	153	37.0
	Never	249	60.1
How often do other members of the household sleep in mosquito nets?	Every time	2	0.4
	Sometimes	155	37.4
	Never	257	62.1
How often do you check for holes/repair mosquito nets?	Every time	1	0.2
	Sometimes	74	17.9
	Never	339	81.9
How often do you use mosquito-repellent coils in your house?	Every time	0	0.0
	Sometimes	0	0.0
	Never	414	100
How often do you use an anti-mosquito spray in your house?	Every time	0	0.0
	Sometimes	83	20.0
	Never	331	80.0
How often do you clean/cut bushes around your house?	Every time	7	1.7
	Sometimes	303	73.2
	Never	104	25.1
How often do you drain stagnant water near your house?	Every time	0	0.0
	Sometimes	194	46.9
	Never	220	53.1
How often do you wear clothes that cover all your body during the night?	Every time	13	3.1
	Sometimes	341	82.4
	Never	60	14.5

## Discussion

The study indicated that 40.9% of the blood films evaluated over 7 years for the presence of malaria parasites were positive. This outcome was similar to a study carried out in the Kola Diba Health Center (40.9%) (Alelign et al., 2018). It was also observed that the overall malaria prevalence was much lower (82.4%) compared to Hallaba (Tefera, 2014) and the East Shewa Zone (82.8%) in the Oromia Region (Gemechu et al., 2015). In contrast, this study showed a higher infection rate (16.34%) than studies carried out in the Dembecha Health Center, Northwest Ethiopia (Haile et al., 2020), Metema Hospital (17%), Ataye (8.4%) in North Shoa (Feleke et al., 2018), and Felegehiwot Referral Hospital (5.0%) in Bahir Dar (Yimer et al., 2017). Such discrepancies might be due to differences in malaria detection methods and laboratory staff members’ abilities to find and identify malaria parasites. Additionally, malaria prevention and control operations may vary from one region to another. Differences in the demography, economic activity, altitude variation, study period, health institute accessibility, community awareness of vector control, and insecticide-treated net (ITN) and indoor residual spraying (IRS) coverage might have an impact on the prevalence of the disease.

The difference in the average annual prevalence of malaria within the study period might be due to differences in climatic conditions. A greater focus on malaria prevention and control measures by various responsible bodies, increased community awareness on the use of ITNs, insecticide spraying, drainage systems for mosquito breeding sites, and global climate change may all be contributing factors to the variation in malaria prevalence.

The highest number of malaria cases coming after heavy rainfall (from June to September) might be due to the rainfall creating a suitable environment for the breeding of *Anopheles* mosquitoes which transmit the disease. Those differences in malaria prevalence based on seasonality are in line with studies conducted in different parts of Ethiopia (Gemechu et al., 2015; Legesse et al., 2015; Hawaria et al., 2019). It has also been underlined that climatic variables such as rainfall, temperature, and altitude are important drivers of malaria dynamics by affecting both malaria parasites and vectors directly or indirectly (FMOH, 2010; Alemu et al., 2011; Sena et al., 2014).

The greater prevalence of *P. falciparum* in this study agreed with the national data and other related research conducted in Ethiopia (FMOH, 2010; Alemu et al., 2012; Amenu, 2014; Ali and Animut, 2019). However, this outcome disagrees with a prior report from Jimma Town that stated that *P. vivax* is more common (Alemu et al., 2011). This study also found that male patients had a greater positivity rate for malaria (54.2%) than female patients in our retrospective study, which is in line with studies conducted in many Ethiopian localities (Alemu et al., 2012; Amenu, 2014; Hawaria et al., 2019; Dabaro et al., 2020). Such a higher incidence rate in male patients may be due to the fact that men frequently engage in outside activities where the risk of mosquito bites is high (Kenea et al., 2016). On the contrary, in the case of the 4-month prospective study (March to June 2022), female patients were more affected (55.1%) than male patients which is similar to a study conducted in the Bahir Dar Health Center (Derbie and Mekonnen, 2017). The difference might be due to variations in sample number between the retrospective and 4-month prospective data.

The prevalence of malaria was also influenced by age. Compared to older age groups, it was higher in younger age groups in the retrospective study. The greater prevalence of malaria in children under 5 years old might be due to these patients having less immune system development than the other age groups. Furthermore, its lower prevalence in the older age group (greater than 90 years old) might be due to less engagement in outdoor activities and a low number of participants which reduces the risk of infection.

The analysis of the 4-month study revealed definite monthly variation in the prevalence of malaria. *P. falciparum* infection in the prospective 4-month study was consistent with the findings of the retrospective investigation. The greater understanding (87%) of malaria prevention techniques is due to better information sources about the disease from health professionals and discussions among family members, which is similar to a study conducted in a malaria-endemic area (Amusan et al., 2017). The majority of the respondents (80.9%) stated that malaria spreads through mosquito bites, which was consistent with research from Ghana (Laar et al., 2013) and India (Forero et al., 2014). This is in contrast to a study conducted in the Shashogo District, Ethiopia, which showed that participants had very little understanding of the method of malaria transmission, with only 15.6% of the participants associating mosquitoes with the disease (Fuge et al., 2015). This study showed more awareness (80.9%) of the association of mosquitoes with the disease.

Nearly all the participants correctly identified the major signs and symptoms of malaria, which is similar to a study carried out in Karachi (Arshia et al., 2013) and additional investigations carried out in Ethiopia (Legesse et al., 2007; Andargie et al., 2013). It is crucial to know mosquito habits, resting and breeding locations, and feeding times to utilize ITNs correctly and avoid malaria. According to the research participants’ observations of mosquito breeding grounds, 266 (64.3%) of them connected mosquitoes to standing water, marshy areas (110, 26.6%), discarded items (23, 5.6%), and discarded materials (23, 5.6%) which is similar to a study conducted in the Shashogo District, Southern Ethiopia (Fuge et al., 2015). Nevertheless, a study conducted in Tepi, southwest Ethiopia, showed that the majority of the community members (96.4%) were aware that mosquitoes breed in stagnant water (Fuge et al., 2015).

Regarding knowledge of interventions for indoor and outdoor prevention and vector control, there was more awareness than the reported rates from Myanmar (Zay and Shwe, 2013). The percentage of respondents who believed that using ITN aids in malaria prevention was lower than in other studies conducted in Ethiopia (Abrar et al., 2017; Flatie and Munshea, 2021). This might be due to the shortage of available ITNs and the economic problems of the people in the study area. Although 96.9% of the total respondents agreed that malaria is a major concern in the study area, only 58.5% sleep under a mosquito net during the night as a method of preventing themselves from mosquito bites. Despite knowledge of ITNs as a protective measure, several participants did not know that fully covering the body at night can prevent the transmission of malaria. Similar findings were reported from a study conducted in Nigeria but the prevalence of wearing of long-sleeved clothes was higher in the Nigerian study than in our study (Amusan et al., 2017). This could be due to a lack of awareness about the importance of wearing long-sleeved clothing to protect against *Anopheles* mosquito bites.

The majority of the participants correctly identified children and pregnant women as the two groups of people the most susceptible to malaria. This is consistent with research from related studies carried out in Kenya (Atieli et al., 2011) and Southwestern Ethiopia (Hawaria et al., 2019). In both cases, a significant number of the participants identified children and pregnant women as the most vulnerable population groups to malaria. This is mostly because pregnant women have immune systems that are partially weakened and children have immune systems that are underdeveloped and weak, making them more susceptible to the disease than adults.

This study was conducted in the spatially limited geographical settings of the selected health centers in the Jawi District. The prospective data collection was only over 4 months (March to June 2022) and that of the retrospective study was only 7 years of recorded data.

## Conclusions

The prevalence of malaria in the retrospective study of 7 consecutive years (2015 to 2021) was found to be high. The trend of infection rate was not consistently increasing or decreasing within the study period but it was varied. Higher malaria prevalence was recorded in the less than 5 years age group. *P. falciparum* was recorded as the main parasite infecting the patients in the month of October and was the lowest in April. The prevalence of malaria in the 4-month prospective study (March to June 2022) was also found as high. Female patients and younger age groups were infected more often and the peak of malaria cases was recorded in June. In this study, the overall knowledge, attitude, and practice level of the study population towards malaria were relatively good. However, a substantial proportion of the participants still had misconceptions about the cause, signs and symptoms, modes of transmission, and practices for prevention methods of malaria. Environmental, biological, socioeconomic, and policy changes may have contributed to the fluctuating trend of malaria prevalence seen in the area. In general, to minimize the prevalence of malaria in the Jawi District, the health office should place more emphasis on preventive measures such as health education, primarily regarding the mode of transmission and prevention and control mechanisms.

## Data Availability

The raw data supporting the conclusions of this article will be made available by the authors, without undue reservation.
